# Utilization and responsiveness of the asthma control test (ACT) at the initiation of therapy for patients with asthma: a randomized controlled trial

**DOI:** 10.1186/1471-2466-12-14

**Published:** 2012-03-26

**Authors:** Mohamed S Al Moamary, Ahmed G Al-Kordi, Mohammed O Al Ghobain, Hani M Tamim

**Affiliations:** 1 College of Medicine, King Saud bin Abdulaziz University for Health Sciences, P.O. Box 84252, Riyadh 11671, Saudi Arabia; 2 Faculty of Medicine, American University of Beirut, Beirut, Lebanon

**Keywords:** Asthma, Asthma control test, Guidelines, Global initiative for asthma, Saudi initiative for asthma, Control

## Abstract

**Background:**

The aim of this study was to assess the responsiveness of the asthma control test (ACT) to detect changes at the initiation of therapy and its utilization in the initiation of asthma treatment.

**Methods:**

This study was designed as a randomized clinical trial conducted in a primary care setting. The subjects were asthma patients who had not received controller therapy for at least two months. The patients were randomized into two groups: The Saudi Initiative for Asthma (SINA) group and the Global Initiative for Asthma (GINA) group. Treatment in the SINA group was initiated at step1 when the ACT scores ≥ 20, step 2 when the score between16-19, and step 3 when the score < 16 began at step 3. The GINA group patients were started on step 2 when they had persistent asthma symptoms or step 3 when they had severely uncontrolled disease.

**Results:**

Forty-five patients were analyzed in each group. The improvement in ACT score after treatment initiation was significantly higher when the SINA approach was used (2.9 in the SINA group compared to 1.7 in the GINA group (*p *= 0.04)). The improvement in FEV_1 _was 5.8% in the SINA group compared to 3.4% in the GINA group (*p *= 0.46). The number of patients who achieved asthma control at the follow-up visit and required no treatment adjustment was 33 (73.3%) in the SINA group and 27 (60%) in the GINA group (*p *= 0.0125).

**Conclusion:**

The ACT was responsive to change at the initiation of asthma treatment and was useful for the initiation of asthma treatment.

**Trial Registration number:**

ISRCTN31998214

## Background

The Global Initiative for Asthma (GINA) has evolved management from being based on a severity index to the concept of achieving asthma control [1-4]. It has adopted a five-step approach to control asthma, where each step represents a different treatment option with increasing efficacy. The five-step approach is designed to maintain control with the least amount of medication [1,5]. For the initiation of treatment, the GINA recommended step 2 for most treatment naïve patients with persistent symptoms, while step 3 was recommended for severely uncontrolled disease [1]. The National Asthma Education and Prevention Program (NAEPP) is another major guideline that utilized asthma severity categorization when initiating treatment in treatment-naïve patients or in those with newly diagnosed asthma [6]. These different approaches were based on a consensus of experts' opinions, as there was insufficient available evidence.

The Saudi Initiative for Asthma management (SINA) was created by the Saudi Thoracic Society which was adopted and customized from the GINA, the NAEPP, and the available local literature [7,8]. The SINA panel has reached a consensus of using the Asthma Control Test (ACT) score to simplify the initiation and adjustment of asthma therapy, as there are variations in the qualifications of health professionals dealing with asthma [6]. The ACT is a validated, short, easy to use, and self-administered instrument used to assess asthma control [8]. It consists of five items that cover a patient's activity limitations, shortness of breath, frequency of night symptoms, use of rescue medication and a rating of overall control of the disease over the past 4 weeks [9,10]. The score of the ACT is the sum of five questions, where each is scored from 1 (worst) to 5 (best), leading to a maximum best score of 25. A score ≥ 20 indicates controlled asthma, scores from 16 to 19 indicate partly controlled asthma, and scores < 16 indicate uncontrolled asthma [11]. In addition to its availability in Arabic, it is a valuable tool that is responsive to changes in patient clinical status over time when used for treatment maintenance and adjustment [6,8,12-14]. Therefore, the objective of this study was to assess the utilization and responsiveness of the ACT at the initiation of asthma therapy in a primary care setting.

## Methods

This study was a randomized clinical trial conducted in asthma patients who presented at primary care centers belongs King Abdulaziz Medical City, Riyadh, Saudi Arabia between 21 September and 12 November 2011. These primary care centers were operated independent of the main hospital and open to patients who has medical records at the center. The inclusion criteria included the following: an age above 12 years, a diagnosis of asthma, and literacy, as patients had to answer the ACT without assistance. Patients were excluded if they had used controller therapy for the two months prior to the initial presentation in order to ensure that our findings are contaminated by a prior controller treatment. Controller therapy was defined as the use of inhaled corticosteroids, leukotriene modifiers, and/or long-acting bronchodilators agents. This study was approved by the Institutional Review Board of the King Abdullah International Center for Medical Research (RC10-091). It was also registered in the International Standard Randomized Controlled Trial Number Register with the number ISRCTN31998214.

### Intervention

**Figure 1:**
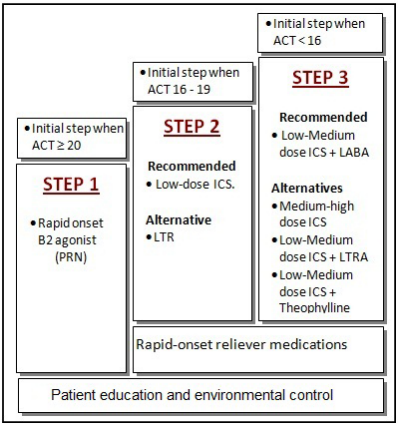
**Initiation of therapy based on the Saudi Initiative for Asthma**.

Patients were randomized to receive their initial treatment based on either the SINA approach (Group A, Figure 1) or the GINA approach (Group B). More specifically, block randomization was carried out with a block size of 4. To avoid selection bias, the random numbers generated were kept in closed opaque envelopes to assure allocation concealment. Following obtaining the written consent from each patient for participation in this study, a research nurse collected each patient's data, which included basic vital signs, a baseline spirometer reading, ACT score and basic demographic data (disease duration, education level, respiratory symptoms, exacerbation and hospital admissions, smoking history, and pulmonary function tests). Peak expiratory flow (PEF) was obtained at the initial and follow-up visits [15]. The measurement of forced expiratory volume in one second (FEV_1_) was performed by a spirometer, as per American Thoracic Society standards [16]. Primary care physicians attended a half-day workshop presented by the authors (MA and AA) on SINA or GINA approaches. An Arabic version of the ACT has been used which was available from the ACT website [14,17,18]. Patients who were assigned to the SINA approach received their initial treatment based on their ACT score [6]. Patients with an ACT score ≥ 20 started with step 1, patients with ACT scores 16-19 started with step 2, and patient with scores less than 16 started with step 3. Patients allocated to group B commenced with step 2 based on the GINA recommendations for persistent asthma symptoms or step 3 when they had severely uncontrolled disease [1]. The physicians of the patients allocated to the GINA approach were blinded to the result of the ACT results. Both guidelines recommended short-acting beta 2 agonists for step 1 and low-dose inhaled steroids for step 2. Although both guidelines recommended the introduction of LABA at step 3, there was a difference in the dose of inhaled steroids where the GINA recommended a low dose of inhaled steroids and SINA recommended a low-medium dose of inhaled steroids. Therefore, to avoid any variation, step 3 was unified to be the combination of LABA with low dose inhaled steroids for both groups. Patients received an educational session for their asthma that included an explanation of the nature of the disease, the importance of compliance, the features of an asthma attack, and inhaler technique. A follow-up visit was offered to the patients four weeks later to assess their level of asthma control using the aforementioned ACT score, and treatments were adjusted accordingly [1,6]. Patients were advised to return to their physicians between visits if they felt that their asthma was not under control.

### Outcome

The primary outcome was measured by changes in the mean FEV_1_, PEF, and ACT scores. More specifically, the proportion of controlled patients in each of the two arms was compared. Any treatment adjustments were also noted at the follow-up visit.

### Sample size and statistical analyses

Sample size calculation, we estimated the controlled patients to be 30% and thus, a sample size of 45 is needed in each arm to have an 80% power to detect a difference of 30%, at an alpha level of 0.05. The data were entered into a Microsoft Excel spreadsheet, which was then transferred into the Statistical Package for Social Sciences (SPSS) program, which was used for data cleaning, management, and analyses. The difference in outcome between the first visit and the follow-up visit was calculated, and then patients with a significant response were flagged. Descriptive analyses were carried out by calculating the number and percent for categorical variables and mean and standard deviation for continuous data. The inferential statistics for the comparison between the two groups was carried out using the Chi-square test for categorical variables, and the *t*-test for continuous variables. The association between the changes in the different measures considered (FEV_1_, PEF, and ACT) between baseline visit and follow-up visit was done by calculating the Pearson correlation coefficient. A *p*-value less than or equal to 0.05 was accepted as statistically significant. All randomized patients were included in the analysis, as per the intention-to-treat principle.

## Results

**Figure 2:**
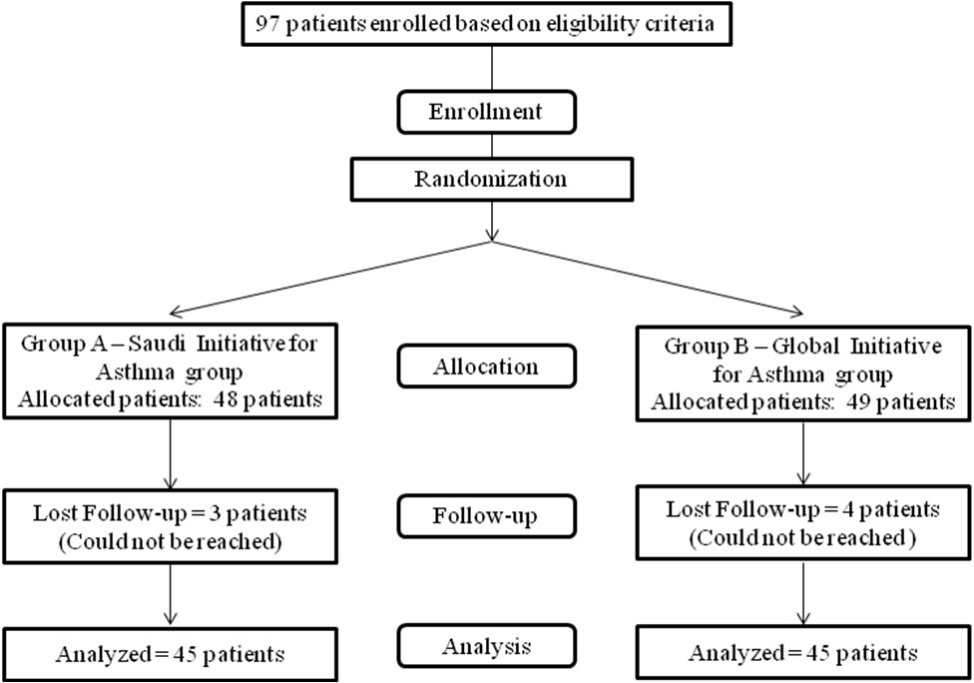
**Enrollment and allocation of patients in the study**.

Ninety-eight patients were recruited for this study (Figure 2). Forty-five patients in each of the two groups (SINA and GINA) completed the study and were analyzed. The baseline characteristics of the two groups were comparable (Table 1). Although the initial PFM and FEV_1 _values were lower in the SINA group than the GINA group, this difference did not reach statistical significance. Table 2 shows the treatment responses of patients assigned to each group. Although there was more improvement in the mean FEV_1 _score of the SINA group (5.8%) than the GINA group (3.4%), this difference did not reach statistical significance (*p *= 0.46). In contrast, the improvement in PEF was higher in the GINA group compared to the SINA group, but this difference also did not reach statistical significance (*p *= 0.803). The improvement in the ACT score after treatment initiation was significantly higher in the SINA group compared to the GINA group (2.9 compared to 1.7 (*p *= 0.04)).

**Table 1:** Baseline characteristics for the Saudi Initiative for Asthma group and the Global Initiative for Asthma group

Characteristics	**SINA**^**a **^**group**	GINA^b ^group	*p *value
Number of patients	45	45	
Age, years (± SD)	42.9 (± 13.4)	42.3 (± 12.6)	0.840
Gender			
- Male, No. (%)	14 (31.1%)	19 (42.2%)	0.274
- Female, No. (%)	31 (68.9%)	26 (57.8%)	
Body mass index, No. (± SD)	32.6 (± 7.4)	34.0 (± 7.3)	0.370
Disease duration, years (± SD)	10.9 (± 9.3)	10.1 (± 8.6)	0.630
Respiratory Symptoms			
- Cough, No. (%)	27 (60.0%)	22 (48.9%)	0.290
- Shortness of breath, No. (%)	10 (22.2%)	15 (33.3%)	0.239
- Wheezing, No. (%)	7 (15.6%)	9 (20.0%)	0.581
Asthma attacks in the past 4 weeks, No. (%)	1.5 (± 1.1)	1.8 (± 1.5)	0.378
Initial visit Peak Flow meter, value (± SD) (L/min)	343.1 (± 129.5)	362.9 (± 148.8)	0.143
Initial visit Forced Expiratory volume in 1 second, Percentage of normal (± SD)	81.4% (± 17.1)	84.3% (± 16.2)	0.269
Initial visit asthma control test score, score (± SD)	17.6 (± 4.5)	17.8 (± 3.6)	0.776

^a ^SINA: Saudi Initiative for Asthma; ^b ^GINA: Global Initiative for Asthma

**Table 2:** Performance of patients assigned to either the Saudi initiative for asthma or Global initiative for asthma approaches after treatment initiation

Characteristics	**SINA**^**a **^**group**	GINA^b ^group	*p*-value
**Forced expiratory volume in 1 second**			
- First visit--No. (± SD)	81.4% (± 17.1%)	84.3% (± 16.2%)	0.269
- Follow-up visit--No. (± SD)	87.2 (± 14.7%)	87.7 (± 17.6)	0.697
- Difference between two visits--No. (± SD)	5.8% (± 12.8)	3.4% (± 13.9)	0.46
**Peak flow meter**			
- First visit--No. (± SD)	343.1 (± 129.3)	362.9 (± 148.8)	0.503
- Follow-up visit--No. (± SD)	389 (± 120.5)	423.1 (± 116.6)	0.182
- Difference between two visits--No. (± SD)	46.84 (63.3%)	60.7 (± 77.8)	0.803
**Asthma control test score**			
- First visit--No. (± SD)	17.6 (± 4.5)	17.8 (± 3.6)	0.776
- Follow-up visit--No. (± SD)	20.5 (± 3.5)	19.5 (± 3.3)	0.43
- Difference between two visits--No. (± SD)	2.9 (± 3.1)	1.7 (± 2.9)	0.04

^a ^SINA: Saudi Initiative for Asthma; ^b ^GINA: Global Initiative for Asthma

Although both groups showed similarities in categorization based on the initial ACT scores (table 1), the follow-up visit revealed that the SINA group contained 32 controlled asthmatics (71.1%), 9 partially controlled asthmatics (20.0%), and 4 uncontrolled asthmatics (31.1%), while the GINA group contained 26 controlled asthmatics (57.8%), 14 partially controlled asthmatics (31.1%), and 5 uncontrolled asthmatics (11.1%) (*p *= 0.185). The correlation between the difference in FEV_1 _and that of the ACT scores between the initial and follow-up visits was found to be statistically significant, with a correlation coefficient of 0.21 (*p *= 0.05). Although there was a positive correlation between the changes in FEV_1 _and PFM (correlation coefficient = 0.19), the change was not significant (*p *= 0.07). Finally, there was a very weak positive correlation between the changes in the PFM and ACT (correlation coefficient = 0.06, *p *= 0.6).

**Figure 3:**
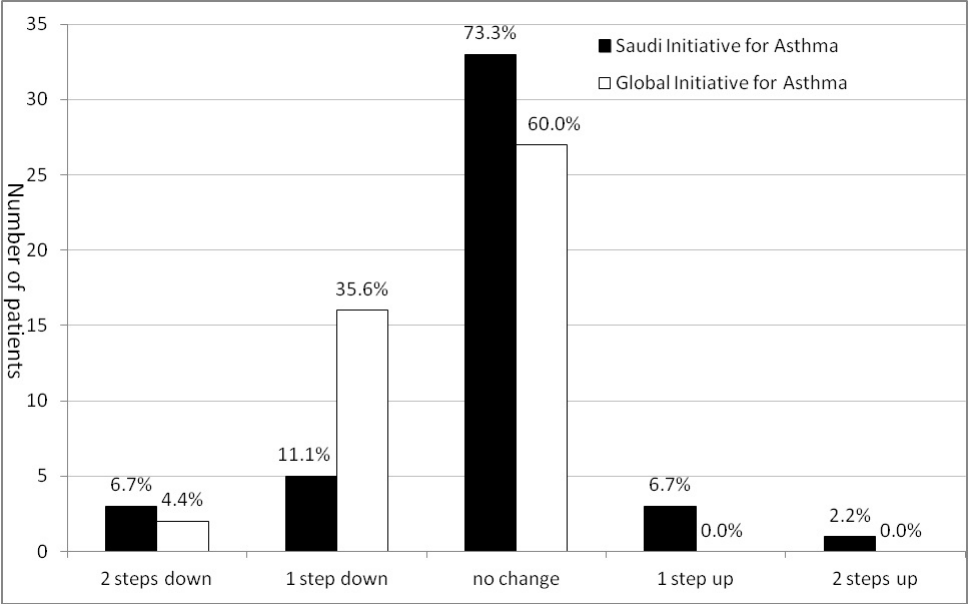
**Follow-up visit step adjustments for patients assigned to either the Saudi initiative for asthma or Global initiative for asthma approaches**.

Figure 3 showed steps adjustments for patients assigned to either approach. There were 33 (73.3%) patients in the SINA group who achieved control at the follow-up visit and required no treatment adjustment, compared to 27 (60%) in the GINA group (*p *= 0.0125). Eight (17.8%) patients in the SINA group required a step down in therapy, and 4 required a step up in treatment at the follow-up visit. In comparison, 18 patients in the GINA group required a step down, while no patients required a step up.

## Discussion

This study showed that the ACT was responsive to changes at the initiation of asthma treatment [6]. It has also showed the usefulness of the ACT score for the initiation of asthma treatment compared to the GINA approach. Despite ample amount of evidence that supports the use of the ACT for treatment adjustment, a unique feature of this study is the presentation of new evidence supporting the utilization of the ACT in making an initial asthma treatment decision. The initiation of asthma treatment has evolved from being based on severity index to achieving asthma control by recommending step 2 for most treatment naïve patients with persistent symptoms and step 3 for severely uncontrolled disease. Both the GINA and the NAEPP approaches require the cumulative experience of healthcare practitioners to make the appropriate clinical judgment. While the severity index and the ACT both use common items related to asthma symptoms, such as nocturnal symptoms, the use of reliever inhalers and the effect of asthma on daily activities, in their classification strategies, the ACT lacks any pulmonary function measurement. On the other hand, a unique characteristic of the ACT is the adoption of a simple 5-item Likert scale that is different from the complex scale adopted in the severity index. Therefore, there are similarities in the items covered in the two instruments, but a lack of pulmonary function measurement in the ACT. The ACT was utilized in this study because it is a standardized objective tool that avoids the variability in the practitioner's experience and qualifications. It has also been utilized by both the SINA and the GINA and validated in the Arabic language [10,13]. Due to the lack of any other validated gold-standard tool for asthma treatment initiation, the GINA approach was included in the study protocol as a benchmark. Moreover, the GINA has received increasing global acceptance as a guideline for asthma management.

Utilizing the initial ACT score to determine the appropriate treatment in this study has led to an improvement of 2.9 units, better than the 1.7 unit improvement observed when treatment was determined by physician judgment based on the GINA approach (*p *= 0.04). Though the improvement in FEV_1 _was better in the patients who followed the SINA approach, this difference did not reach statistical significance. This lack of statistical significance could be related to an inadequate sample size to assess a positive response of this outcome. The conflicting results obtained regarding the improvement in PEF in the GINA group may be related to the poor correlation of PEF with the ACT and pulmonary function when used to assess asthma control [19,20]. An interesting study from Greece utilized the ACT to assess disease control after treatment initiation in naïve patients, showed an average response of 1.73 units, which is similar to the response we observed in the group that used the GINA standards [21]. The uncontrolled asthma status present in the previous study based on initial ACT was found to be statistically correlated with a higher fractional exhaled nitric oxide (FeNO) and positively correlated with pre-bronchodilator FEV_1_. Moreover, the change in the ACT between the two visits was significantly correlated with the change in the FEV_1 _and FeNO in that study [22]. The scanty data regarding the correlation of the ACT with different parameters at the initiation of treatment are supported by studies regarding the utilization of the ACT to assess asthma control. Recent studies have also shown that a score of less than 19 on the ACT has a 66% sensitivity in detecting uncontrolled asthma and serves as an objective measure of control that correlates well with the GINA [22-24]. Another study from Hong Kong showed that an ACT score ≤ 20 correlated better with treatment decision than PEF and FeNO. Their finding revealed a sensitivity of 70.5% and specificity of 76.0% [20]. Although FEV_1 _is the main objective physiological measurement of asthma control, the ACT was also found to correlate well with lung function and inflammation [20,25]. Moreover, the ACT was found to be a useful tool for detecting poorly controlled asthma to optimize asthma control [26]. These studies have shown that the ACT was at least equivalent to pulmonary function or FeNO in assessing asthma control or making a treatment adjustment. This has a practical implication for treatment adjustment due to the unavailability of spirometry or FeNO in primary care settings, especially in developing countries.

We observed a non-significant trend toward achieving control upon follow-up when the ACT was utilized to determine a treatment plan. Nevertheless, the SINA group patients showed significant stability in treatment plan upon follow-up when compared to the GINA group patients (73.3% vs. 60%). This was supported by the fact that 40% of those treated with the GINA approach required a step down in treatment upon follow-up, compared to 17.8% of those who treated with the SINA approach. Commencing treatment with the appropriate dose would enhance compliance and minimize the side effects of medications. On the other hand, 8.9% of the patients who followed the SINA required a step-up in treatment compared to none in the GINA group, a finding that may indicate an inadequate initial treatment.

Finally, it is worth mentioning a few limitations and concerns related to this study. The utilization of the ACT as an objective measure for initiating asthma therapy is independent of the practitioners' clinical judgment. In contrast, the knowledge of those practitioners who utilized the GINA approach in this study may have been augmented by the pre-study workshop, possibly contaminating the results in that group. This issue was discussed during the preparation of the study protocol, and the authors felt that it was inappropriate to deny the practitioners' placement in the GINA arm due to ethical considerations. In day-to-day practice, most practitioners have variability in their knowledge and experience and may not have the opportunity for dedicated education sessions. Therefore, it is an area for future research to challenge our findings in general practice. Another limitation was the use of set ACT score limits of 16 and 19 for decisions regarding the appropriate initial treatment step. Due to the lack of evidence identifying ACT reference scores for treatment initiation, these numbers were extrapolated from studies that assessed asthma control to make decisions about treatment adjustment and maintenance. Defining the ACT categories that determine initial treatment is another area that needs to be challenged to support its suitability [25,27].

## Conclusions

We believe that despite the aforementioned limitations and concerns, this study can be considered a pilot project that showed the ACT to be responsive to change at treatment initiation and showed usefulness for the initiation of asthma treatment compared to the GINA approach.

## Abbreviations

ACT: Asthma control test; SINA: Saudi Initiative for Asthma; GINA: Global Initiative for Asthma; NAEPP: National Asthma Education and Prevention Program; PEF: Peak expiratory flow; FEV_1_: Forced expiratory volumes in one second; FeNO: Fractional exhaled nitric oxide.

## Competing interests

The authors declare that they have no competing interests.

## Authors' contributions

MSAM: primary author, study design, analysis, and writing of the manuscript. MOAG: study design, data collection, and writing of the manuscript. AGAK: data collection, approvals, and writing of the manuscript. HMT: study design, randomization, data analysis and manuscript writing. All authors read and approved the final manuscript.

## Support

This study was supported by a grant from the King Abdullah International Center for Medical Research.

## Pre-publication history

The pre-publication history for this paper can be accessed here:

http://www.biomedcentral.com/1471-2466/12/14/prepub
